# Supplementary material for: Contribution of Proteins to the Latin American Diet: Results of the ELANS Study
Source: Nutrients. 2023 Jan 28;15(3):669. doi: 10.3390/nu15030669 (PMC9920156; doi:10.3390/nu15030669)
Supplement: Supplementary file 1 [file nutrients-15-00669-s001.zip › nutrients-2123437-supplementary.pdf]

Supplementary material

Contribution of proteins to the Latin American diet: Results of the ELANS study

Marianella Herrera-Cuenca, Martha Cecilia Yépez García, Lilia Yadira Cortés Sanabria, Pablo Hernández, Yaritza Sifontes, Guillermo Ramírez, Maura Vásquez, Georgina Gómez, María Reyna Liria-Domínguez, Attilio Rigotti, Mauro Fisberg, Irina Kovaslkys, Maritza Landaeta-Jiménez.

Table of contents

Supplemental Table S1. Mean and 95% CI for animal and plant protein intake by ELANS countries.

Supplemental Table S2. Mean daily protein intake (g/day) by socio-economic class and educational level, by protein source, by and country.

Supplemental Table S3. Mean daily protein intake (g/day) by socio-economic level and educational level, by protein source, and by ELANS countries.

Supplemental Table S1. Mean and 95% CI for animal and plant protein intake by ELANS countries.

**Country**	**n**	**Animal**			**Plant**		
		Mean	95% CI		Mean	95% CI	
ELANS	9218	51.3	51.0	51.7	27.1	26.9	27.2
Argentina	1266	59.7	58.7	60.7	26.2	25.7	26.7
Brazil	2000	54.2	53.3	55.1	25.0	24.6	25.3
Chile	879	38.9	38.1	39.8	28.6	28.0	29.3
Colombia	1230	53.9	53.0	54.7	26.5	26.1	26.8
Costa Rica	798	38.8	37.7	39.9	29.2	28.5	29.9
Ecuador	800	56.5	55.3	57.6	29.2	28.7	29.7
Peru	1113	46.1	45.3	47.0	31.9	31.4	32.4
Venezuela	1132	54.2	53.4	55.1	23.4	23.0	23.9

Supplemental Table S2. Mean daily protein intake (g/day) by sex and age, by protein source, and by ELANS countries.

	Country	n	Total group (g/d)		Sex (g/d)				Age group (g/d)							
					Male		Female		15 - 19.9		20 - 34.9		35 - 49.9		50 - 65	
			Mean	SD	Mean	SD	Mean	SD	Mean	SD	Mean	SD	Mean	SD	Mean	SD
TOTAL SOURCE	ELANS	9218	78.8	23.1	86.7	23.8	71.5	19.8	80.6	22.3	81.6	23.7	77.9	23.3	73.7	21.3
	Argentina	1266	85.9	23.6	95.9	24.1	77.7	19.6	91.3	22.3	86.1	23.8	85.8	24.4	83.0	22.2
	Brazil	2000	79.2	26.7	89.1	28.3	70.5	21.8	85.1	27.6	82.2	28.4	78.0	25.8	72.3	22.5
	Chile	879	67.5	19.0	75.3	20.4	60.2	14.2	65.5	15.3	69.5	18.8	68.7	22.6	64.4	15.8
	Colombia	1230	80.4	19.9	85.9	20.1	75.0	18.3	82.0	19.2	82.3	19.3	79.5	19.3	77.7	21.6
	Costa Rica	798	68.0	22.5	77.3	23.4	59.0	17.2	67.4	17.7	74.0	24.1	66.0	22.4	59.6	19.2
	Ecuador	800	87.4	21.6	94.7	21.1	80.2	19.6	85.7	19.3	91.8	21.0	85.9	22.5	81.1	21.6
	Perú	1113	79.5	20.0	86.7	20.2	73.1	17.6	79.7	19.6	82.4	21.1	78.8	19.5	73.6	17.1
	Venezuela	1132	77.7	19.4	83.7	20.0	72.1	16.9	80.7	18.3	80.5	20.6	76.0	18.9	72.0	16.4
ANIMAL SOURCE	ELANS	9218	51.3	18.2	55.8	19.2	47.2	16.2	51.9	18.3	53.3	18.5	50.8	18.0	48.1	17.1
	Argentina	1266	59.7	18.3	66.2	18.8	54.3	15.9	62.4	18.9	59.8	18.3	59.8	18.8	57,9	17.2
	Brazil	2000	54.2	20.7	60.0	22.3	48.9	17.6	57.6	21.8	55.9	22.0	53.6	19.9	50.0	18.0
	Chile	879	38.9	13.1	42.6	14.3	35.6	10.9	37.4	10.2	39.6	13.3	40.2	15.0	37.2	11.4
	Colombia	1230	53.9	15.3	57.3	15.5	50.6	14.4	54.5	14.1	55.8	15.2	53.0	14.9	51.8	16.1
	Costa Rica	798	38.8	15.2	42.9	16.3	34.8	12.8	38.8	13.7	43.0	16.2	36.9	14.0	33.2	13.7
	Ecuador	800	56.5	16.3	60.8	15.9	52.2	15.6	55.2	16.0	59.7	16.2	55.3	15.9	52.0	16.3
	Perú	1113	46.1	14.5	49.3	15.3	43.3	13.2	45.1	15.2	48.5	15.0	45.3	13.9	42.5	12.8
	Venezuela	1132	54.2	15.1	58.3	15.7	50.4	13.5	56.1	13.6	56.8	16.1	52.6	14.8	49.7	13.1
PLANT SOURCE	ELANS	9218	27.1	8.7	30.0	9.2	24.4	7.2	28.3	8.6	27.6	8.9	26.8	8.8	25.5	8.0
	Argentina	1266	26.2	8.4	29.3	8.9	23.6	7.0	28.5	9.0	26.3	8.8	25.9	8.3	25.2	7.6
	Brazil	2000	25.0	8.7	28.5	9.2	21.9	6.9	27.1	8.9	25.9	9.2	24.5	8.6	22.6	7.3
	Chile	879	28.6	9.4	32.3	9.9	25.2	7.3	28.4	8.2	29.5	9.9	28.5	9.9	27.7	8.4
	Colombia	1230	26.5	6.9	28.1	7.2	24.9	6.2	27.3	7.1	26.4	6.6	26.6	6.8	26.0	7.4
	Costa Rica	798	29.2	10.0	33.6	10.2	24.9	7.7	28.6	8.8	30.3	10.3	29.5	10.3	27.0	9.6
	Ecuador	800	29.2	7.2	31.4	7.6	27.0	6.1	29.2	6.3	30.2	7.4	28.9	7.8	27.3	6.2
	Perú	1113	31.9	8.5	35.4	8.6	28.8	7.0	33.2	9.5	32.3	8.5	31.9	8.2	30.0	7.8
	Venezuela	1132	23.4	7.4	25.0	7.8	21.9	6.7	24.2	7.3	23.6	7.1	23.3	7.7	22.6	7.5

Supplemental Table S3. Mean daily protein intake (g/day) by socio-economic level and educational level, by protein source, and by ELANS countries.

	**Country**		**Total group** (g/d)		**Socioeconomic class** (g/d)						**Educational level**					
					High		Middle		Low		Basic		Superior		Professional	
		n	Mean	SD	Mean	SD	Mean	SD	Mean	SD	Mean	SD	Mean	SD	Mean	SD
TOTAL SOURCE	ELANS	9218	78.8	23.1	80.5	24.1	79.3	23.2	78.1	22.8	78.1	23.1	80.3	23.3	79.6	21.7
	Argentina	1266	85.9	23.6	86.3	21.0	86.2	23.0	85.7	24.4	86.5	23.8	84.2	23.4	85.3	19.3
	Brazil	2000	79.2	26.7	83.7	30.6	80.6	26.5	77.0	26.0	77.9	26.5	81.4	27.0	79.2	24.6
	Chile	879	67.5	19.0	65.9	15.8	66.4	15.9	68.9	22.0	67.2	19.8	68.5	16.6	67.9	19.3
	Colombia	1230	80.4	19.9	85.6	16.1	82.7	18.9	78.8	20.5	79.2	20.1	82.2	20.3	83.5	17.3
	Costa Rica	798	68.0	22.5	71.8	23.8	69.0	21.9	65.0	22.6	67.7	22.9	69.0	20.7	71.8	19.5
	Ecuador	800	87.4	21.6	90.3	23.9	89.0	22.0	85.5	20.5	85.6	20.2	94.9	26.4	98.2	25.5
	Peru	1113	79.5	20.0	78.8	20.7	78.9	19.0	80.2	20.4	78.1	20.4	79.7	19.8	81.0	20.7
	Venezuela	1132	77.7	19.4	83.0	22.3	80.1	20.6	76.9	18.8	77.2	19.1	81.5	20.5	77.7	19.5
ANIMAL SOURCE	ELANS	9218	51.3	18.2	52.2	18.8	51.8	18.5	50.8	17.8	50.8	18.1	52.0	18.4	52.9	17.3
	Argentina	1266	59.7	18.3	59.8	17.1	60.3	17.9	59.1	18.8	59.9	18.4	59.0	18.1	60.1	16.7
	Brazil	2000	54.2	20.7	58.9	23.4	55.4	20.9	52.0	19.8	52.7	20.1	56.2	21.3	54.9	19.8
	Chile	879	38.9	13.1	39.8	11.9	39.1	11.7	38.6	14.5	38.1	13.3	40.6	11.8	40.3	13.9
	Colombia	1230	53.9	15.3	58.8	10.9	55.7	14.7	52.6	15.7	52.9	15.5	55.4	15.1	56.5	13.9
	Costa Rica	798	38.8	15.2	42.9	15.9	39.6	14.6	35.8	15.5	38.2	15.5	40.3	13.8	44.5	12.8
	Ecuador	800	56.5	16.3	60.0	17.9	57.4	15.8	54.8	16.1	55.2	15.5	60.8	18.7	65.3	18.5
	Peru	1113	46.1	14.5	46.5	15.1	46.0	13.4	46.1	15.0	45.1	14.6	46.2	14.4	48.0	15.2
	Venezuela	1132	54.2	15.1	58.3	16.1	56.5	15.9	53.5	14.8	53.7	14.9	57.0	15.8	54.6	15.5
PLANT SOURCE	ELANS	9218	27.1	8.7	27.5	8.5	27.1	8.5	27.0	8.9	27.0	8.8	27.7	8.8	26.1	8.1
	Argentina	1266	26.2	8.4	26.7	7.1	25.8	8.4	26.5	8.6	26.5	8.5	25.3	8.3	25.0	6.8
	Brazil	2000	25.0	8.7	24.7	8.0	24.9	8.3	25.1	9.2	25.3	9.3	25.0	8.1	24.1	8.2
	Chile	879	28.6	9.4	26.1	7.7	27.4	8.0	30.3	10.5	29.2	9.6	27.5	8.7	27.7	9.1
	Colombia	1230	26.5	6.9	26.4	7.5	26.9	6.7	26.2	7.0	26.3	6.8	26.8	7.1	26.7	7.3
	Costa Rica	798	29.2	10.0	28.7	9.4	29.3	9.9	29.2	10.6	29.4	10.3	29.1	8.5	27.1	8.5
	Ecuador	800	29.2	7.2	28.4	7.5	29.7	7.7	29.1	6.8	28.9	6.8	30.7	9.7	31.0	8.1
	Peru	1113	31.9	8.5	30.7	8.9	31.5	8.3	32.7	8.4	31.6	8.6	32.2	8.6	30.8	7.6
	Venezuela	1132	23.4	7.4	24.4	8.0	23.6	7.0	23.3	7.4	23.4	7.6	24.4	7.3	23.0	6.6
